# Awareness of Antimicrobial Resistance and Antibiotic Use Among Patients Attending the Outpatient Department of a Tertiary Care Hospital

**DOI:** 10.7759/cureus.105029

**Published:** 2026-03-11

**Authors:** Mohammed Thaha S, Usha Krishnan K, Thasneem Banu S

**Affiliations:** 1 Medicine, Madras Medical College, Chennai, IND; 2 Microbiology, Madras Medical College, Chennai, IND

**Keywords:** antibiotics, antibiotic stewardship, antimicrobial resistance, community knowledge, healthcare-seeking behavior, irrational antibiotic use, prescription practices, public awareness, safe use of antibiotics, self medication

## Abstract

Introduction: Antimicrobial resistance (AMR) is an increasing global public health problem that threatens the effective prevention and treatment of infectious diseases. Inappropriate antibiotic use, including self-medication, premature discontinuation of therapy, and misuse for viral infections, plays a major role in the development and spread of AMR. The increasing occurrence of multidrug-resistant organisms and the limited availability of effective antibiotics have further worsened this problem.

Aim: To assess the knowledge, attitudes, and practices related to antibiotic use and antimicrobial resistance among patients attending the outpatient department of a tertiary care hospital; to identify common factors contributing to inappropriate antibiotic use, such as self-medication and incomplete antibiotic courses; and to determine the association between demographic variables and the knowledge, attitudes, and practices related to antibiotic use and antimicrobial resistance during the study period.

Methodology: This hospital-based cross-sectional study was conducted among adult outpatients of a tertiary care hospital over four months. Consecutive sampling was conducted in 259 participants (137 were females [52.9%] and 122 males [47.1%]). Data were collected using a pre-validated questionnaire assessing knowledge, attitudes, and practices related to antibiotic use and antimicrobial resistance. The data were analyzed using IBM Corp. Released 2025. IBM SPSS Statistics for Windows, Version 31. Armonk, NY: IBM Corp. with descriptive statistics and Pearson chi-square tests, considering p < 0.05 as statistically significant.

Results: Awareness of antibiotics was reported by 241 participants (93.1%), but only 93 (35.9%) correctly identified that antibiotics treat bacterial infections only, while 132 (51.0%) believed they treat both viral and bacterial infections. Awareness of antimicrobial resistance (AMR) was reported by 151 (58.3%), and 127 (49.0%) correctly understood antibiotic resistance as resistance of microorganisms. Regarding attitudes, 174 participants (67.2%) reported that taking antibiotics without a prescription is unacceptable. Antibiotics should not be stopped once symptoms improve was reported by 134 (51.7%) . In terms of practices, 49 (18.9%) reported self-medication, and 78 (30.1%) reported not completing prescribed antibiotic courses. Pearson Chi-square analysis showed that education level was significantly associated with knowledge and attitudes toward antibiotic use and antimicrobial resistance (χ² = 36.437, p ≤ 0.05). Gender showed a significant association with beliefs regarding stopping antibiotics early (χ² = 12.850, p = 0.005), while age and residence were not significantly associated (p > 0.05).

Conclusion: The study highlights a clear gap between awareness, knowledge, and actual practices related to antibiotic use among outpatient attendees. Although most participants had heard of antibiotics, misconceptions regarding their indications and antimicrobial practices, with self-medication and incomplete antibiotic courses remaining common. Educational level emerged as the most influential factor affecting antibiotic-related knowledge and attitudes. These findings emphasize the need for targeted educational programs, improved patient counseling during outpatient visits, and stricter enforcement of prescription-only antibiotic policies to promote rational antibiotic use and reduce antimicrobial resistance.

## Introduction

Antimicrobial resistance (AMR) is a major and escalating global public health concern that threatens the effective prevention and treatment of infectious diseases. The World Health Organization (WHO) identifies AMR as one of the top ten global health threats, as it progressively diminishes the efficacy of antibiotics and other antimicrobial agents [1]. As resistance increases, infections that were once easily treatable now require longer hospital stays and more expensive medications and are associated with increased morbidity and mortality.

Inappropriate and irrational use of antibiotics in the community remains one of the most significant contributors to the development and spread of AMR [2]. Common practices such as self-medication, use of leftover antibiotics, premature discontinuation of prescribed courses, and obtaining antibiotics without a valid prescription are widely reported, particularly in low- and middle-income countries [3,4]. The CDC (Centers for Disease Control and Prevention) update report says that at least 28% of outpatient antibiotic prescriptions were deemed unnecessary. Furthermore, misconceptions regarding antibiotic effectiveness have led to their frequent misuse for viral infections such as the common cold, influenza, and non-specific febrile illnesses [5]. 

Healthcare systems worldwide are witnessing a rise in multidrug-resistant (MDR) organisms, particularly in hospital settings. Resistant pathogens are increasingly implicated in severe infections such as hospital-acquired pneumonia, bloodstream infections, and sepsis, where standard treatment regimens often fail, resulting in poor clinical outcomes [6]. Surveillance reports indicate a worrying increase in resistance to commonly used antibiotics, including beta-lactams, fluoroquinolones, and carbapenems [7].

The challenge of AMR is further intensified by the slow pace of new antibiotic development. Recent global analyses highlight that the antibiotic development pipeline remains insufficient, with most newly approved agents belonging to existing drug classes and offering limited benefit against resistant organisms [8]. Consequently, preserving the effectiveness of current antibiotics through responsible use is of critical importance.

Several studies conducted across diverse populations have demonstrated significant gaps in knowledge, attitudes, and practices related to antibiotic use. Inadequate awareness has been reported not only among the general public but also among students, caregivers, pharmacy trainees, and healthcare workers in training [9,10]. Educational level, cultural beliefs, access to healthcare, and exposure to reliable information strongly influence antibiotic-related behaviors [11].

Assessing community-level awareness and practices is essential for designing effective educational and behavioral interventions aimed at reducing antibiotic misuse. Understanding local patterns of antibiotic use can inform targeted awareness programs and support national and global AMR containment strategies [12]. In this context, the present study aims to assess the awareness of antimicrobial resistance and antibiotic use among patients attending the outpatient department of a tertiary care hospital, with the goal of identifying knowledge gaps and guiding future interventions to combat AMR.

## Materials and methods

Study design and setting

This was a hospital-based, cross-sectional study conducted in the Outpatient Department (OPD) of Rajiv Gandhi Government General Hospital, a tertiary care hospital. The study was carried out over a period of four months, from August to November 2025.

Ethical considerations

The study protocol was reviewed and approved by the Institutional Ethics Committee prior to the initiation of data collection (Approval No.: 76052025). Written informed consent was obtained from all participants before enrolment, and confidentiality of participant information was strictly maintained throughout the study.

Study population and sample size

A total of 259 adult participants were included in the study by the consecutive sampling method. 

Inclusion and exclusion criteria

Adults aged 18 years and above attending the outpatient department for routine medical consultations were eligible for inclusion. Healthcare workers and medical or paramedical students were excluded from the study to minimize professional knowledge-related bias.

Data collection tool (questionnaire)

Data were collected using a structured, pre-validated questionnaire designed to assess participants’ knowledge, attitudes, and practices (KAP) regarding antibiotic use and antimicrobial resistance. The questionnaire was reviewed and validated by subject experts prior to its use to ensure clarity, relevance, and content validity. It consisted of closed-ended questions covering key domains such as awareness of antibiotic indications, understanding of antimicrobial resistance, attitudes toward antibiotic adherence, and common antibiotic-use practices. The questionnaire was administered by the principal investigator. To ensure accurate comprehension, each question and its response options were explained to participants in the local language. Completed questionnaires were reviewed immediately for completeness and consistency, and any missing or unclear responses were clarified at the time of data collection.

Data management

All completed questionnaires were coded and entered into Microsoft Excel™ (Redmond, USA) for data compilation. Data quality checks were performed regularly, and inconsistent or illogical entries were verified and corrected before analysis.

Statistical analysis

The finalized dataset was exported to IBM Corp. Released 2025. IBM SPSS Statistics for Windows, Version 31. Armonk, NY: IBM Corp. for statistical analysis. Descriptive statistics, including frequencies, percentages, means, and standard deviations, were used to summarize the data. The distribution of the continuous variable (age) was assessed using histogram inspection. Age was approximately normally distributed and therefore presented as mean ± standard deviation. Categorical variables were summarized using frequencies and percentages. Associations between demographic variables and knowledge, attitudes, and practices regarding antibiotic use and antimicrobial resistance were analyzed using the Pearson chi-square test. A p-value <0.05 was considered statistically significant.

## Results

Demographic characteristics

A total of 259 participants were included, of whom 137 (52.9%) were females, and 122 (47.1%) were males (Table 1). The majority were aged 18-25 years (34.7%), followed by 36-45 years (27.0%) and 26-35 years (18.9%). The mean age was 33.18 ± 11.76 years. Most participants had completed undergraduate (47.5%) or postgraduate education (41.7%). Private-sector employees constituted 47.1%, while students accounted for 30.9%. Most participants resided in urban areas (71.4%).

**Table 1:** Demographic information of the participants

	Variable	Value	Percentage
Gender	Female	137	52.9
	Male	122	47.1
Age	18–25 years	90	34.7
Mean 33.18	26–35 years	49	18.9
Std. Deviation 11.76	36–45 years	70	27.0
	46–55 years	34	13.1
	56–65 years	13	5.0
	>65 years	3	1.2
Educational Level	Uneducated	5	1.9
	Middle school	2	0.8
	High school	21	8.1
	Under Graduate	123	47.5
	Post graduate	108	41.7
Occupation	Private sector	122	47.1
	Students	80	30.9
	Homemakers	25	9.7
	Government sector	20	5.7
	Self-employed	4	1.6
	Unemployed	8	3.1
Location	Urban	185	71.4
	Sub-urban	27	10.4
	Rural	47	18.1

Knowledge of antibiotics and antimicrobial resistance

Most participants (93.1%) had heard of antibiotics (Table 2). Only 35.9% correctly identified that antibiotics are effective solely against bacterial infections, while 51.0% incorrectly believed they are effective against both viral and bacterial infections. Antibiotic side effects were acknowledged by 69.1% of participants. Awareness of antimicrobial resistance (AMR) was reported by 58.3%. A correct understanding of antibiotic resistance as resistance of microorganisms was observed in 49.0% of participants, whereas 31.7% incorrectly believed that the human body becomes resistant.

**Table 2:** Status of knowledge regarding antibiotic use and AMR of the respondents

Question	Options	n	Percentage (%)
Have you heard the term “antibiotics”?	Yes	241	93.1
	No	18	6.9
What do you think antibiotics treat?	Both viral & bacterial infections	132	51.0
	Only bacterial infections	93	35.9
	Only viral infections	8	3.1
	Don’t know	26	10.0
Do you think antibiotics have side effects?	Yes	179	69.1
	Not sure	54	20.8
	No	26	10.0
Have you heard the term AMR / antibiotic resistance?	Yes	151	58.3
	No	108	41.7
What do you understand by “antibiotic resistance”?	Disease-causing organisms becoming resistant	127	49.0
	Body becomes resistant to antibiotics	82	31.7
	Don’t know	50	19
Do you think antibiotic resistance is a serious public health issue?	Yes	150	57.9
	Not sure	80	30.9
	No	29	11.2

Attitudes toward antibiotic use

The majority of participants (67.2%) felt that antibiotic use without a doctor’s prescription is unacceptable (Table 3), and 69.5% believed antibiotics should not be shared with others. About half of the participants reported that antibiotics should not be discontinued once symptoms improve (51.7%) and acknowledged their role in preventing AMR (51.4%). Despite this, self-use of antibiotics for common illnesses such as colds, coughs, fevers, and sore throats was commonly reported. Most participants could not recall the antibiotic name; amoxicillin was the most frequently mentioned among those who could.

**Table 3:** Level of attitude about antibiotic use and AMR of the respondents

Question	Options	n	Percentage (%)
Do you believe it is acceptable to take antibiotics without a doctor’s prescription?	No	174	67.2
	Sometimes	48	18.5
	Yes	25	9.7
	Don’t know	12	4.6
Can antibiotics be shared with others who have similar symptoms?	No	180	69.5
	Yes	39	15.1
	Don’t know	40	15.4
Can we stop taking antibiotics once symptoms improve even if the full course is not completed?	No	134	51.7
	Yes	45	17.4
	Sometimes	44	17.0
	Don’t know	36	13.9
Do you think you have a role in preventing antimicrobial resistance?	Yes	133	51.4
	No	30	11.6
	Don’t know	96	37.1
Would you like to receive more information about appropriate antibiotic use?	Don’t know	6	2.3
	No	50	19.3
	Yes	203	78.4

Antibiotic use practices

Over half of the participants (53.7%) had used antibiotics in the preceding six months (Table 4). Self-medication with antibiotics was reported by 18.9% of participants. Non-completion of prescribed antibiotic courses was reported by 30.1%, most commonly due to symptomatic improvement. Antibiotics were primarily obtained through prescriptions, with smaller proportions sourced from pharmacies or non-medical advice. Regarding disposal, leftover antibiotics were mainly thrown into the dustbin or stored for future use, and only a very small number followed proper disposal methods or shared them with others.

Association between demographic variables and KAP on antibiotic use and AMR

Analysis of independent factors against KAP regarding antibiotic use and AMR of the participants is illustrated in Table 4.

**Table 4:** Analysis of independent factors against KAP regarding antibiotic use and AMR of the participants

Factor	Associated Variables (Significant)	Pearson Chi-Square (χ²)	df	p-value	Significance
Gender	Belief about not stopping antibiotics early	12.850	3	0.005	Significant
	Desire for more information	7.968	2	0.019	Significant
	All other comparisons	–	–	>0.05	Not significant
Education level	Heard of antibiotics	14.734	4	0.005	Significant
	Knowledge of what antibiotics treat	36.437	12	<0.001	Significant
	Awareness of AMR	36.437	12	<0.001	Significant
	Understanding of antibiotic resistance	18.171	4	0.001	Significant
	Belief about stopping antibiotics early	25.758	12	0.012	Significant
Occupation	Heard of antibiotics	93.617	39	<0.001	Significant
	Heard of AMR	27.189	13	0.012	Significant
	Other comparisons	–	–	>0.05	Not significant
Location	Heard of antibiotics	7.281	2	0.026	Significant
	All other comparisons	–	–	>0.05	Not significant

Gender showed significant associations with certain attitudes toward antibiotic use. Female participants more frequently reported that antibiotics should not be stopped once symptoms improve compared with males (χ² = 12.85, p = 0.005). Females also expressed greater interest in receiving further information about appropriate antibiotic use compared with males (χ² = 7.968, p = 0.019).

Education level showed significant associations with several knowledge-related variables. In general, better knowledge and appropriate attitudes were more commonly observed among participants with undergraduate and postgraduate education compared with those with lower education levels. Awareness of antibiotics was higher among undergraduate and postgraduate participants (χ² = 14.734, p = 0.005). Correct knowledge regarding what antibiotics treat was also more frequently observed among undergraduate and postgraduate participants (χ² = 36.437, p < 0.001). Similarly, awareness of antimicrobial resistance and correct understanding of antibiotic resistance as resistance of microorganisms were more commonly reported among undergraduate and postgraduate participants (χ² = 36.437, p < 0.001; χ² = 18.171, p = 0.001). Appropriate beliefs regarding not stopping antibiotics early were also more common among participants with undergraduate and postgraduate education (χ² = 25.758, p = 0.012).

Location showed a significant association only with having heard of antibiotics, whereas its association with knowledge, attitudes, and practices related to antibiotic use was not significant (p > 0.05). A greater proportion of urban participants reported having heard the term "antimicrobial resistance" (114) compared with rural (22) and suburban participants (15); however, this association was not statistically significant (χ² = 3.476, p = 0.176). Similarly, awareness of antibiotics was higher among urban participants (177) compared with rural (40) and suburban participants (24).

Occupation was significantly associated only with having heard of antibiotics and awareness of antimicrobial resistance, while all other comparisons were not significant. Awareness of antimicrobial resistance was most frequently observed among participants working in the private sector (72) and among students (54), while smaller numbers were observed among other occupational groups. Awareness of antibiotics was also highest among participants in the private sector (114) and students (77).

## Discussion

The present study assessed the knowledge, attitudes, and practices related to antibiotic use and antimicrobial resistance among patients attending the outpatient department of a tertiary care hospital. Although awareness of antibiotics was high among participants (93.1%), important gaps were identified in correct knowledge, particularly regarding indications for antibiotic use and the concept of antimicrobial resistance.

In this study, only 93 (35.9%) out of 259 participants correctly identified that antibiotics are effective exclusively against bacterial infections, while 132 (51.0%) participants believed that antibiotics are useful for both bacterial and viral infections, which is similar to a KAP study on antibiotic use and resistance in which the majority (93%) of the respondents defined antibiotics incorrectly [13]. Similar misconceptions have been reported in studies from other parts of India and low- and middle-income countries, where antibiotics are frequently used for self-limiting viral illnesses such as the common cold and fever [4,13]. Such misunderstandings contribute significantly to inappropriate antibiotic consumption and the development of antimicrobial resistance.

Awareness of antimicrobial resistance was moderate, with 151 (58.3%) of the participants reporting familiarity with the term, and 150 (57.9%) participants acknowledged it as a serious public health problem. However, 127 (49.0%), less than half correctly understood antibiotic resistance as resistance developed by microorganisms rather than by the human body which is similar to a study stating that 52% of participants expressed misconception that humans become resistant to antibiotics [14]. This misconception has been consistently observed in previous studies and highlights a critical knowledge gap that can undermine public health messaging on AMR [15,16]. Such a misunderstanding of the concept of resistance may lead to inappropriate antibiotic practices. About half of the participants (133, 51.4%) believed that they have a role in preventing AMR, and 78.4 of them wanted to receive more information about appropriate antibiotic use.

Disapproval regarding consuming antibiotics without a prescription and sharing antibiotics was registered by 174 (67.2%) and 180 (69.5%) of the participants, respectively. Despite such positive attitudes toward antibiotic use, unsafe practices persisted. Nearly one-fifth (49, 35.25%) of participants reported self-medication with antibiotics. Studies highlight that the prevalence of antimicrobial self-medication is high and is associated with inappropriate antibiotic use [17].

Regarding practices, only 139 (53.7%) participants had used antibiotics in the past six months; among them, 49 (35.25%) consumed antibiotics with a doctor’s prescription. However, 78 (56.11%) of the participants did not complete the full course, mainly because they felt better before finishing the medication. This is in contrast to a study that reports that 90.14% recognized the importance of completing antibiotic courses and 32.16% admitted they would stop taking antibiotics once they felt better [18]. Similar patterns have been reported in multiple Indian and international studies, where symptomatic improvement is a leading reason for premature discontinuation of antibiotics [19,20]. Such practices contribute directly to the selection and spread of resistant organisms.

Education level showed the strongest associations and was significantly linked with awareness of antibiotics, knowledge of what antibiotics treat, awareness of antimicrobial resistance (AMR), understanding of antibiotic resistance, understanding of antibiotic use, and beliefs regarding stopping antibiotics early (p ≤ 0.05). Participants with undergraduate (UG) and postgraduate (PG) educational qualifications demonstrated significantly better awareness and understanding of antibiotics and AMR compared to those with lower educational levels. Educational status and urban residence were significantly associated with better awareness and understanding of antibiotics and AMR in the present study. Inappropriate practices, including self-medication and premature discontinuation of therapy, were significantly more common among younger participants and those with lower educational levels (p < 0.05). This finding is consistent with earlier research demonstrating that higher education levels and improved access to healthcare information are associated with more appropriate antibiotic-related knowledge and behaviors [21,22]. These findings emphasize the need for targeted educational interventions focusing on populations with lower educational attainment and those residing in rural areas.

Overall, the findings of this study reveal a discrepancy between knowledge, attitudes, and actual practices related to antibiotic use. While awareness levels were moderate and attitudes generally favorable, inappropriate practices such as self-medication and incomplete treatment courses remain prevalent. These findings underscore the need for continuous public education, stricter enforcement of prescription-only antibiotic policies, and incorporation of AMR awareness programs at the community and outpatient levels. Strengthening patient-provider communication during outpatient visits may play a key role in improving antibiotic use behaviors and mitigating the growing threat of antimicrobial resistance.

Limitations

This study has certain limitations. Being a single-center, hospital-based cross-sectional study, the findings may not be generalizable to the wider community. Data were collected using a self-reported questionnaire, which may be subject to recall bias and social desirability bias. Despite these limitations, the study provides valuable insights into antibiotic use and AMR awareness among outpatient populations in a tertiary care setting.

## Conclusions

The present study highlights that although general awareness of antibiotics among outpatient attendees was high, significant gaps persist in correct knowledge regarding their appropriate use and antimicrobial resistance. Misconceptions about antibiotic effectiveness for viral infections, moderate awareness of AMR, and unsafe practices such as self-medication, premature discontinuation of antibiotic therapy, and improper disposal of leftover antibiotics were commonly observed. While attitudes toward antibiotic use were largely positive, these did not consistently translate into appropriate practices. The findings underscore the need for targeted community-based educational interventions, strengthened patient counseling during outpatient visits, and stricter enforcement of prescription-only antibiotic policies to promote rational antibiotic use and curb the growing threat of antimicrobial resistance.
